# Artificial intelligence for children with attention deficit/hyperactivity disorder: a scoping review

**DOI:** 10.3389/ebm.2025.10238

**Published:** 2025-04-24

**Authors:** Bo Sun, Fei Cai, Huiman Huang, Bo Li, Bing Wei

**Affiliations:** ^1^ Department of Neonatology, General Hospital of Northern Theater Command, Shenyang, Liaoning, China; ^2^ Post-Graduate College, China Medical University, Shenyang, Liaoning, China

**Keywords:** artificial intelligence, attention deficit/hyperactivity disorder, machine learning, deep learning, review method

## Abstract

Attention deficit/hyperactivity disorder is a common neuropsychiatric disorder that affects around 5%–7% of children worldwide. Artificial intelligence provides advanced models and algorithms for better diagnosis, prediction and classification of attention deficit/hyperactivity disorder. This study aims to explore artificial intelligence models used for the prediction, early diagnosis and classification of attention deficit/hyperactivity disorder as reported in the literature. A scoping review was conducted and reported in line with the PRISMA-ScR (Preferred Reporting Items for Systematic Reviews and Meta-Analyses Extension for Scoping Reviews) guidelines. Out of the 1994 publications, 52 studies were included in the scoping review. The included articles reported the use of artificial intelligence for 3 different purposes. Of these included articles, artificial intelligence techniques were mostly used for the diagnosis of attention deficit/hyperactivity disorder (38/52, 79%). Magnetic resonance imaging (20/52, 38%) were the most frequently used data in the included articles. Most of the included articles used data sets with a size of <1,000 samples (28/52, 54%). Machine learning models were the most prominent branch of artificial intelligence used for attention deficit/hyperactivity disorder in the studies, and the support vector machine was the most used algorithm (34/52, 65%). The most commonly used validation in the studies was k-fold cross-validation (34/52, 65%). A higher level of accuracy (98.23%) was found in studies that used Convolutional Neural Networks algorithm. This review provides an overview of research on artificial intelligence models and algorithms for attention deficit/hyperactivity disorder, providing data for further research to support clinical decision-making in healthcare.

## Impact statement

At present, artificial intelligence is a hot topic, but it still needs to be developed in the medical field, especially in pediatric clinical research. We believe that the researchability of artificial intelligence is sufficient. As we know, in the medical field, early diagnosis and identification of a certain clinical disease is crucial for clinical doctors, and the emergence of artificial intelligence is likely to bring tremendous assistance to clinical diagnosis and treatment work. In this study, we conducted scope evaluation according to the PRISMA-ScR guidelines, and mainly summarized AI models and algorithms for diagnosis, prediction, and classification of attention deficit/hyperactivity disorder. The hope is to provide clinical decisions that support future research in healthcare.

## Introduction

Attention-deficit/hyperactivity disorder (ADHD) is a neurodevelopmental disorder caused by the interaction of genetic and environmental factors that has a worldwide prevalence of 7.2% in children [1, 2]. ADHD is characterized by a persistent and impairing pattern of inattention and/or hyperactivity/impulsivity, about 60% of children with ADHD have symptoms that persist into adulthood [3], and 89% of ADHD patients are accompanied by mental illness, representing a significant public health problem [4]. Therefore, early diagnosis of ADHD is critical to enable early intervention and treatment.

At present, the diagnosis of ADHD mainly relies on the judgment of psychiatrists, based primarily on reports from parents and teachers, behavioral observations, and clinical interviews, which are sensitive to subjective biases [5, 6]. Existing studies have shown that ADHD is a highly heterogeneous disease involving multiple etiological and risk factors, with different clinical characteristics, development process and outcome, which brings diagnostic challenges to clinicians, and false positive diagnosis or misdiagnosis may occur in clinical practice [7, 8]. It has been shown that a significant association between disease and trait does not necessarily imply that it can be used for disease prediction. Neuroimaging plays a vital role in the study of brain function by visualizing the structure and activity of the brain, allowing researchers to understand how different brain regions are involved in various cognitive and behavioral processes [9]. The brains of children with ADHD are different in terms of structure and function, and these differences are also associated with neurocognitive performance. Structural magnetic resonance imaging (sMRI), functional MRI (fMRI), resting-state fMRI (rs-fMRI) and diffusion tensor imaging (DTI) were used to characterize the etiology and phenotype of ADHD from different dimensions [10]. Genome-wide association studies have also revealed several variants in ADHD [11, 12]. In addition, other studies have attempted to use electrocardiogram (ECG) signals [13], eye tracking [14], physiological signals, wearable device data [15], and exercise data to help diagnose ADHD.

Artificial intelligence (AI) is a technology with great potential in medicine, machine learning (ML) is a powerful tool for making critical decisions by analyzing large data sets such as social behavior patterns and various health conditions, deep learning (DL) is a branch of ML [16]. Many neurological diseases are identified based on subjective diagnostic criteria. Neuroimaging is a promising objective diagnostic tool. The task of ML is to model the relationship between features extracted from imaging data and individual labels in the data set, which can be used for new or invisible data sets. It creates broad prospects for disease diagnosis, prognosis and management in health care and enriches personalized medicine [17]. With the increasing popularity of AI models, AI technology has achieved satisfactory results in the diagnosis of brain-related diseases such as Alzheimer’s disease, Parkinson’s disease, autism spectrum disorder (ASD) [18], and ADHD is no exception. AI can assist in ADHD diagnosis, classification, prognosis, treatment prediction, and the development of new therapeutic drugs.

A large number of articles have been published on AI technologies for ADHD. Several reviews were conducted to summarize previous studies; however, they had the following limitations: First, they focused on studies of ADHD diagnosis with machine learning methods using MRI data [19]; Second, they focused on describing the efficacy of ML or DL models in the diagnosis, classification, or prediction of ADHD, without describing in detail the characteristics of the AI algorithms used [20]. The available literature lacks a review that provides an overview of the features of the AI algorithms used in ADHD. Thus, this review aims to explore the characteristics of AI models used for the diagnosis, prediction and classification to aid scientists advance research on this field.

## Materials and methods

### Overview

In this scoping review, we conducted a systematic literature search that reviewed research involving the use of AI for ADHD prediction, classification, and diagnosis. To ensure the transparency and reliability of this study, the literature search was conducted according to the Preferred Reporting Items for Systematic Review and Meta-Analysis Protocols Extension for Scoping Reviews (PRISMA-ScR) guidelines [21]. The protocols used in the scoping review are detailed in the following sections.

### Search strategy

#### Search sources

Two authors (Bo Sun and Fei Cai) conducted an independent search in February 2025 and screened abstracts and full texts, which were finally checked by the corresponding author (Bing Wei). During this period, we searched four online databases, including MedRXiv, BioRXiv, PubMed, and Science Direct. The search focused on both medical and computer science databases.

#### Search terms

We used the following items as keywords: (“artificial intelligence” OR “machine learning” OR “deep learning” OR “supervised learning” OR “unsupervised learning” OR “reinforcement learning”) AND (“attention-deficit/hyperactivity disorder”) AND (diagnosis* OR detect* OR predict* OR screen*). For more information on the exact search terms used to search each database, see Multimedia Supplementary Appendix S1.

### Eligibility criteria

The studies included in this review mainly concerned AI technologies for ADHD diagnosis and risk prediction. In other words, we focus on AI models related to ADHD diagnosis. The search was limited to original journal research articles in English. We excluded articles (i.e., literature reviews, dissertations) outlining AI approaches to ADHD as well as studies based purely on clinical trials and experimental studies. Inclusion criteria include: (1) AI technology; (2) the goal to diagnose or screen for ADHD; (3) participants are children only; (4) the data is publicly available. Exclusion criteria include: (1) inadequate details in terms of AI models; (2) same raw data; (3) inappropriate article types (e.g., case reports, reviews, papers, proposals, conference abstracts, editorials, generic manuscripts, and reviews).

### Study selection

Articles selected from each database were charted on Microsoft Excel. At the same time, we imported all the retrieved articles into the EndNote software, and the duplicate check function was used to remove duplicate studies. Titles and abstracts were carefully selected and screened, and articles were searched for full text reading if they met the inclusion criteria. Any disagreements were resolved through discussion among the investigators. To measure agreement between investigators, we calculated the Cohen kappa [22], where the screening result for title and abstract was 0.976, while the screening result for full text was 0.82. We documented the inter-investigator agreement matrix in Multimedia Supplementary Appendix S2.

### Data extraction

The investigators performed the data extraction process using a pre-designed standardized form (Multimedia Supplementary Appendix S3). The extracted data included: (1) author, country, and year; (2) the age, number and health status of the participants; (3) the source, setting, and availability of the data used by AI; (4) algorithms, types, and features of AI models; (5) outcomes of AI diagnosis of ADHD.

## Results

### Search results

**FIGURE 1:**
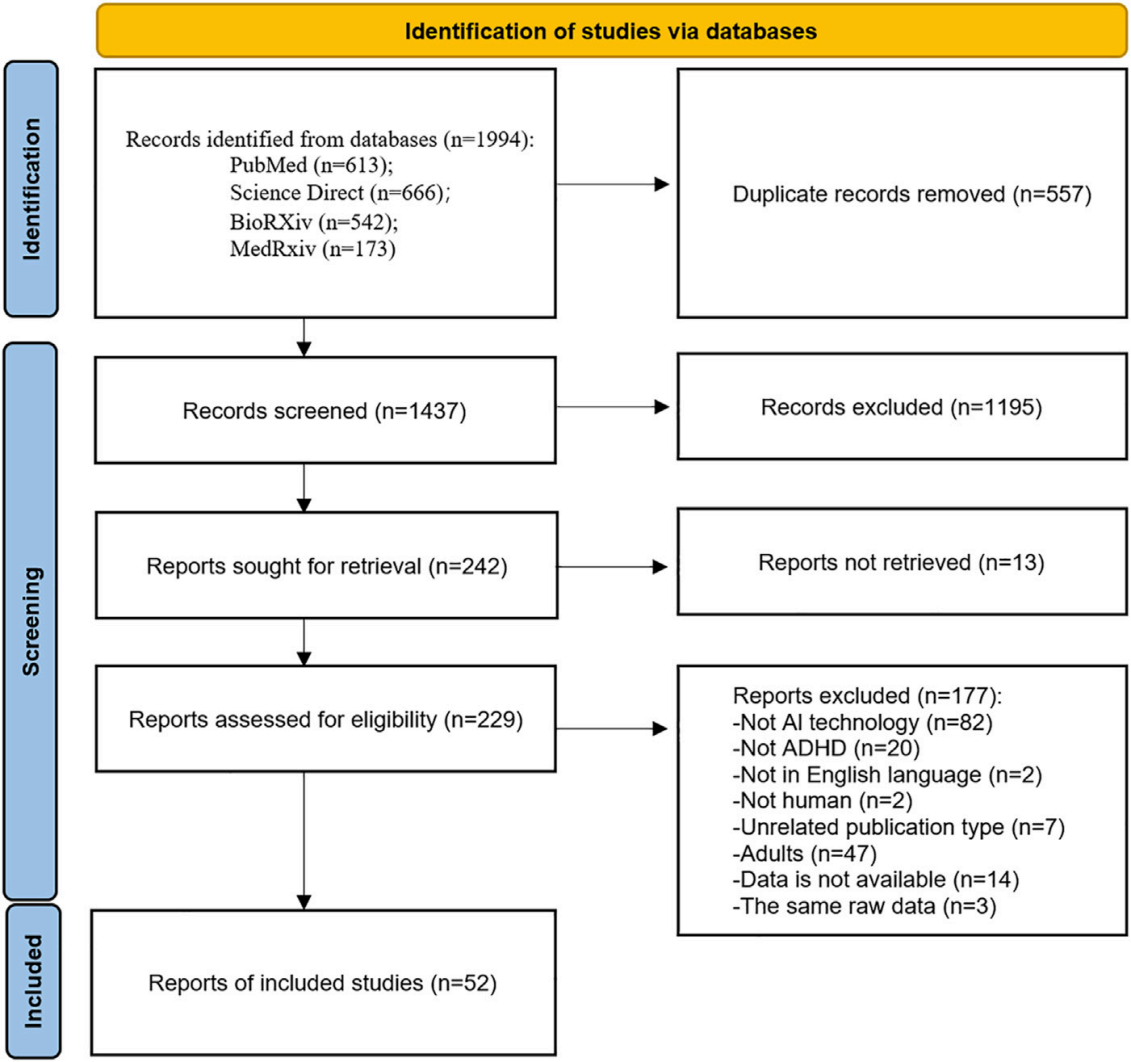
PRISMA-ScR flowchart of the study selection.

We preliminarily identified 1994 articles using four open online databases: PubMed (n = 613), Science Direct (n = 666), BioRXiv (n = 542), and MedRxiv (n = 173). After that, we excluded 557 duplicate articles. Of the remaining studies, 1,195 articles were removed after title and abstract screening. In addition, 13 articles were not searchable, so 229 articles were included in the full-text screening. As shown in Figure 1, after reviewing the full text, we excluded 177 articles for a variety of reasons. A total of 52 articles met our inclusion criteria and were included in this scoping review.

### Main characteristics of the included articles

Characteristics of the included studies were shown in Table 1. All of the studies we included were published in peer-reviewed journals (52/52, 100%). Eligible studies were published between 2012 and 2025, mainly in China (16/52, 31%), followed by Korea (9/52, 17%). The number of participants mentioned in the included studies ranged from 10 to 238,696. Of these, 33 studies reported the proportion of female participants, ranging from 2% to 50%. Furthermore, 88% (46/52) of the included studies only recruited participants with ADHD, and 12% (6/52) of the studies included participants with other medical conditions. Multimedia Supplementary Appendix S4 showed the detailed characteristics of the included studies.

**TABLE 1:** Characteristics of the included studies (n = 52).

Characteristics	Studies n (%)	References
Publication type		
Journal articles	52 (100)	[12, 13, 15, 23–28], [29–71]
Year of publication, n (%)		
2025	1 (1.9)	[26]
2024	9 (17.3)	[26, 63, 64, 66–71]
2023	8 (15.4)	[35, 42, 47, 50]
2022	7 (13.5)	[23, 27, 33, 39, 45, 60, 61]
2021	4 (7.7)	[12, 13, 24, 38]
2020	4 (7.7)	[25, 37, 44, 52]
2012–2019	19 (36.5)	[28–32, 34, 36, 40–44, 48, 54–56, 58, 59, 62]
Country of publication		
China	16 (31)	[12, 24, 28, 29, 39, 46, 54, 59, 61, 64, 66, 68–71]
Korea	9 (17)	[15, 25, 30, 47, 49, 50, 55, 58]
United States	7 (13)	[23, 32, 38, 40, 41, 52, 57]
Canada	2 (4)	[31, 43]
Germany	2 (4)	[34, 60]
Spain	3 (6)	[48, 56, 63]
Australia	1 (2)	[53]
Denmark	1 (2)	[67]
Iran	1 (2)	[33]
Israel	1 (2)	[37]
India	1 (2)	[42]
Italy	1 (2)	[62]
Japan	1 (2)	[44]
Minnesota	1 (2)	[36]
Singapore	1 (2)	[13]
Sweden	1 (2)	[27]
Turken	1 (2)	[35]
Türkiye	1 (2)	[51]
United Kingdom	1 (2)	[45]
Number of participants, n (%)		
<99	17 (33)	[25, 30, 34–36, 38, 47–49, 52, 56, 58, 62, 66, 69–71]
100–999	28 (54)	[13, 15, 23, 26, 28, 29, 31, 33, 37, 39–44, 46, 50, 51, 54, 55, 59–61, 63–65, 67, 68]
>1,000	7 (13)	[12, 24, 27, 32, 45, 53, 57]
Gender, range (%)		
Female	2–50	[12, 13, 15, 24, 28, 30, 31, 33, 35–40, 42, 44–47, 52, 55–60, 62–65, 67, 69, 70]
Participants’ health conditions, n (%)		
Only ADHD	46 (88)	[12, 13, 15, 23–31, 34–39], [41–52, 54–60, 62–66, 68–71]
ADHD and OTHERS	6 (12)	[32, 33, 40, 53, 61, 67]

### Characteristics of AI techniques for ADHD

Of the included studies, 76.9% used only ML algorithms, 9.6% used only DL algorithms, and 13.5% applied ML including DL algorithms. In addition, we collated the AI models, algorithms, and methods used in the included ADHD studies. The most commonly used model was support vector machine (SVM, 34/52, 65%), followed by random forest (RF, 17/52,33%). In the 52 studies, AI algorithms were used for 3 different purposes. The most common purposes were early diagnosis (38/52,79%) and risk predictions (10/52, 14%; Table 2). Only 11 studies stated the programming languages used to develop the models, and they were R (5/52, 10%) and Python (6/52,12%). Multimedia Supplementary Appendix S5 showed the characteristics of the AI techniques used in each study.

**TABLE 2:** Types of AI techniques used for ADHD (n = 52 studies).

Types	Studies n (%)	References
AI type		
ML	40 (76.9)	[13, 15, 23–26, 28–34, 36, 38, 39, 41–49, 52–56, 58, 60, 62–65, 67–70]
DL	5 (9.6)	[35, 50, 51, 61, 66]
ML and DL	7 (13.5)	[12, 27, 37, 40, 57, 61, 71]
AI algorithms/models/methods ^a^		
SVM	34 (65)	[12, 13, 23, 24, 28–34, 39–46, 48, 49, 52, 54–60, 62, 63, 67, 68, 70]
RF	17 (33)	[12, 15, 24, 27, 32, 33, 36–38, 40, 42, 45, 53, 63, 69–71]
DT	10 (19)	[13, 31, 32, 38, 52, 53, 63, 68, 69, 71]
Gradient boosting	7 (13)	[15, 26, 27, 33, 63, 69, 70]
K-nearest neighbors (KNN)	7 (13)	[27, 31, 38, 42, 43, 45, 68–70]
AdaBoost	6 (12)	[13, 27, 36, 63, 69, 70]
LR	6 (12)	[27, 32, 38, 42, 43, 45]
Convolutional neural network (CNN)	5 (10)	[12, 35, 51, 56, 59, 61]
Naive bayes (NB)	5 (10)	[12, 35, 51, 59, 61, 63]
Extreme learning machine (ELM)	3 (6)	[30, 54, 55]
Multi-layer perceptron (MLP)	3 (6)	[45, 59, 63]
Neural network (NN)	3 (6)	[37, 40, 63]
Deep-learning neural network (DNN)	2 (4)	[27, 66]
Linear discriminant (LDA)	2 (4)	[32, 47]
Multinomial regression (MR)	2 (4)	[40]
Recurrent neural network (RNN)	2 (4)	[50, 71]
Categorical lasso	1 (2)	[32]
Classification and regression tree (CART)	1 (2)	[70]
Elastic net regularization (EN)	1 (2)	[58]
Partial least squares (PLS)	1 (2)	[40]
Purpose of AI algorithms		
Early diagnosis	38 (79)	[15, 24, 25, 28–43, 47, 49–57, 59–62, 65–67, 70, 71]
Predicting	10 (14)	[12, 23, 26, 27, 44, 45, 58, 63, 64, 68]
Classification	4 (7)	[13, 46, 48, 69]
Programming languages ^b^		
Python	6 (12)	[23, 26, 27, 32, 53, 64]
R	5 (10)	[25, 39, 41, 44, 47]

^a^ Some studies used more than one model.

^b^ Only 9 studies reported the programming languages used to develop the model.

Table 3 showed the different data categories used in the included studies: 38% of the studies (20/52) involved brain imaging, 25% (13/52) included demographic information, 19% (10/52) used electroencephalogram (EEG), and so on. 60% of the included studies used datasets from closed-source (i.e., data collected directly from databases of study participants or clinical settings) and 40% from open-source (i.e., publicly available databases). The numbers of features used to develop the models in the included studies ranged from 3 to 13,585,634. And 25 studies (48%) did not exceed 100 features in developing their model. We provided a detailed description of the number of features and data categories of the included studies in Multimedia Supplementary Appendix S6.

**TABLE 3:** Features and categories of data used in the included articles (n = 52 studies).

Features	Studies n (%)	References
Data category ^a^		
Brain imaging	20 (38)	[23–25, 29–31, 33, 35, 37, 41–43, 46, 54–58, 65, 67]
Demographic information	13 (25)	[28, 29, 37, 38, 41–45, 58, 60, 67]
EEG measurements	10 (19)	[13, 26, 34, 39, 49, 51, 59, 61, 66, 71]
Parent/Teacher report questionnaire	9 (17)	[32, 36, 45, 52, 53, 60, 63, 68, 71]
Neurocognitive features	7 (13)	[36, 37, 44, 50, 52, 60, 62]
Eye tracking	3 (6)	[48, 64, 66]
Genetic characteristics	3 (6)	[12, 25, 58]
Behavioral data	2 (4)	[69, 70]
Wearable data	2 (4)	[15, 40]
Others	3 (6)	[28, 47, 62]
Number of features		
<99	25 (48)	[13, 15, 26, 27, 32–34, 36, 40, 43, 45, 46, 52, 58–60, 62–65, 67–71]
100–999	10 (19)	[23, 29, 30, 38, 42, 47, 54, 56, 57, 66]
>1,000	9 (15)	[12, 24, 25, 31, 41, 49, 51, 53, 55]
Not reported	8 (15)	[28, 35, 37, 39, 44, 48, 50]
Type of data set source		
Closed	31 (60)	[12, 13, 15, 23, 26, 34–40, 44, 49, 50, 52, 56–60, 62–71]
Open	21 (40)	[24, 25, 27–33, 40–42, 44, 46–48, 51, 53–55, 61]

^a^ Many studies used more than one data category.

As shown in Table 4, the included studies used different validation techniques in the development of AI models, mainly of two. Among them, k-fold CV (34/52, 65%) is the more commonly used method. Only 13% of studies (7/52) mentioned confusion matrices, but all 52 studies mentioned performance metrics for AI models. According to statistics, the most commonly used performance measure was accuracy (ACC, 45/52, 87%). In Table 5, 8 studies reported the precision of AI algorithms, ranging from 80% to 95%, with an average of 92.53%; The area under the curve (AUC) in 26 studies ranged from 57.6% to 99.64%, with a mean of 83.77%; The mean ACC of the 45 studies was 83.06%, ranging from 53.2% to 98.23%; 35 studies reported specificities varying between 58.8% and 99.11%, with a mean of 84.08%; The F1-score valued in 11 studies ranged from 48.89% to 95%, with a mean of 85.21%. In addition, the sensitivity of the AI algorithms reported in 35 studies ranged from 33% to 98.24%, with an average of 74.67%.

**TABLE 4:** Validation approaches and performance measures (n = 52 studies).

Validation and statistics	Studies n (%)	References
Validation approach ^a^		
K-fold CV	34 (65)	[13, 24, 27–35, 37–39, 41–43, 45, 46, 48, 51, 53, 57–64, 68–71]
LOOCV	10 (19)	[25, 26, 28, 47, 50, 52, 54, 56, 61, 62]
Not reported	11 (21)	[12, 15, 23, 36, 40, 44, 49, 55, 65–67]
Confusion matrix		
Reported	7 (13)	[35, 36, 50, 51, 53, 61, 63]
Not reported	45 (87)	[12, 13, 15, 23–34], [37–49, 52, 54–60, 62, 64–71]
Performance measures ^b^		
ACC	45 (87)	[12, 13, 23–31, 33–43], [46–50, 52–54, 56–64, 66–71]
Sensitivity	35 (67)	[12, 13, 15, 24, 26–29, 31, 33, 36–42, 44, 46, 47, 50–53, 55–57, 60–64, 67, 69, 71]
Specificity	35 (67)	[12, 13, 15, 24, 26–29, 31, 33, 36–42, 44, 46, 47, 50–53, 55–57, 60–64, 67, 69, 70]
AUC	26 (50)	[12, 15, 24, 25, 27, 29, 32, 35, 38, 40, 45, 46, 48, 49, 53, 54, 58–65, 69, 70]
F1-score	11 (21)	[23, 26, 35, 45, 46, 50, 55, 61, 64, 68, 71]
Precision	8 (15)	[26, 35, 45, 53, 55, 64, 68, 71]
Recall	5 (10)	[35, 45, 55, 69, 71]
False-negative	3 (6)	[36, 48, 50]
False-positive	3 (6)	[36, 48, 50]
Negative predictive value	3 (6)	[15, 27, 33]
Positive predictive value	2 (4)	[15, 33]
Kappa	1 (2)	[40]
J-statistic	1 (2)	[46]
Positive predicted power	1 (2)	[27]
True-negative	1 (2.4)	[36]
True-positive	1 (2.4)	[36]

^a^ Total number does not add up, as many studies used more than one validation method.

^b^ Total number does not add up, as many studies used more than one performance measure.

**TABLE 5:** Overview of performance of AI models.

Performance measures	Results (%), mean (range)
Precision	92.53 (80–95)
AUC	83.77 (57.6–99.64)
ACC	83.06 (53.2–98.23)
Specificity	84.08 (58.8–99.11)
F1-score	85.21 (48.89–95)
Sensitivity	74.67 (33–98.24)

## Discussion

### Principal findings

In this study, we explored the application of AI techniques in the early diagnosis, prediction, and classification of ADHD. We searched articles published from January 2012 to February 2025, and of the 1994 articles retrieved, 52 were eventually included in our scoping review. Over the past 4 years, an increasing number of studies have been published: 9 in 2024, 8 in 2023, 7 in 2022, and 4 in 2021. Tracing its causes, the digital innovation process has stimulated the increasing demand for telemedicine programs, and healthcare systems have increasingly relied on AI technology [72]. In the field of child and adolescent neuropsychiatry, the development and use of online platforms for collecting case histories, demographic, and behavioral information have been steadily increasing [73]. The increase in available data has provided new opportunities for cutting-edge methods such as ML and DL, which used high-dimensional data to build predictive models to capture non-linear relationships across multiple data sources, traditional statistical methods could not achieve [74]. The articles we included focused on AI being used for three purposes in ADHD: early diagnosis, classification, and prediction. None of the included articles were used for other purposes, such as treatment response prediction, prognosis, drug efficacy evaluation, and patient outcomes. Similar to the application of AI in other mental disease, China, the United States and South Korea (32/52, 61.54%) were the countries with largest number of studies related to the use of AI in ADHD.

The data available in the application of AI in ADHD could be roughly divided into the following seven categories: demographic characteristics (gender, age, race, ethnicity, parental education, etc.); parent/teacher report questionnaire; neurocognitive characteristics; brain imaging (fMRI, sMRI, DTI) [20]; genetic data; EEG; eye tracking. Among them, 20 studies included MRI. MRI has demonstrated the possible physiological basis of the disease and is a potential predictor. ML or DL techniques may help identify reliable features and use this as a classification or diagnostic predictor [23]. Zhou, Lin [24] constructed a multimodal ML framework combining Boruta-based feature selection and multi-core learning, integrating sMRI, fMRI and DTI data for early diagnosis of ADHD. Then they used SVM to distinguish ADHD from healthy children. AUC of the model for diagnosing ADHD was 69.8%, and the classification ACC was 64.3%. The reported ACC of existing ADHD classification models varied, with most ranging between 60 and 90% [75]. Despite the success of MRI-based ML models, it has been found that models that incorporated demographic characteristics and/or parent/teacher questionnaires reported higher ACC in classification or diagnosis. One study evaluated parent/teacher ratings of executive function (from BRIEF’s Emergent Metacognition Composite score), behavioral/cognitive measures of executive function, measurements of cortical thickness in frontal subregions, and thickness and volume in the parietal cortex, two demographic characteristics (age and child sex), as well as a complete model with four categories. The results showed that the complete model with all the target features achieved a performance ACC of 0.994 in predicting ADHD diagnosis, with 0.926 derived from parent/teacher reports, which was considered critical in classifying ADHD [76]. ADHD was highly heritable (76% heritability) [77]. There was a study that combined multimodal MRI with candidate genetic data [25], including cortical morphology, diffusivity scalars, resting-state functional connectivity and polygenic risk score from norepinephrine, dopamine and glutamate genes. The integration of candidate single nucleotide polymorphism (SNP) data into the best model did not show a meaningful improvement in ACC. Existing studies of modeling using AI technique have all incorporated MRI diagnostic tools, in fact, it is important to acknowledge that neuroimaging data yields very little power [78]. There is still a need to focus on readily available behavioral/clinical data, including demographic information, subjective symptom ratings, and objective neuropsychological data. Integrated modeling approaches could facilitate the development of new approaches to ADHD classification and treatment. New types of data, such as eye tracking, could also be considered in the future in combination with clinical features.

Traditional ML and DL are two branches of AI. In this review, we investigated the characteristics of AI techniques present in the research. Most studies used ML, and the most commonly used algorithm was SVM (34, 65%), followed by RF (17, 33%). SVM by identifying the optimal hyperplane or by mapping nonlinear data into high-dimensional space using kernel functions to realize classification [79]. Its strength resides in its proficiency in managing small sample sizes, high-dimensional data, and nonlinear datasets efficiently, as exemplified when utilizing EEG to analyze ADHD [26]. Nevertheless, it is hindered by significant computational complexity and a heightened sensitivity to parameter adjustments. Conversely, Based on the voting mechanism of the integrated decision tree (DT), RF is good at processing large-scale multimodal data (such as when applying multi-center imaging and clinical data fusion to characterize ADHD) [80], does not require feature selection and is robust, and RF is known for its ability to perform well in classification and regression tasks [81]. However, the high complexity of the model leads to weak interpretability, and overfitting may occur in extreme cases [82]. The application scenarios of the two in the field of ADHD are significantly different: SVM is suitable for accurate classification tasks with limited data but complex features, while RF is more suitable for mining potential patterns in large-scale data. The sample size of ADHD research data is limited, so SVM is more suitable. DT and logistic regression (LR) are rarely used because they are difficult to cope with the high dimensional, non-linear and heterogeneous characteristics of ADHD data [27].

In contrast, DL was used 12 times (23.1%). K-fold CV was used in 34 (34/52, 65%) studies for AI model testing. In the early days, ML was widely used for its simplicity and high efficiency, owing to its advantages over traditional analytical methods based on mass-univariate statistics, especially considering the inter-correction among regions [16]. DL is a particular subtype of ML which is based on deep neural networks (DNNs). In contrast to ML technology, which requires manual extraction of features during image segmentation, DL employs artificial neural networks (ANNS) that allow direct processing of raw data and are particularly useful in identifying complex patterns in high-dimensional fMRI data to maximize model performance for related tasks [83]. Although there are few DL studies, their results are better than those of ML. There are several issues to be noted, one is the limitation of data volume, due to cross-sample reliability/validity and sensitivity and specificity limitations, ADHD diagnosis is primarily based on parent/teacher reports, neuroimaging is not yet part of the routine diagnosis process of ADHD [84]. Most of the MRI data in the published studies come from public databases, such as ADHD-200, the Study of Cognitive Development in the Adolescent Brain (ABCD), and Autism Brain Imaging Data Exchange (ABIDE), which have limited sample size and limited reproducibility [6, 85], the amount of data that is available is still not enough to meet the needs of DL. Secondly, it is the lack of transparency in the learning and testing process of DL that has led them to be called black boxes, and the interpretability of medical algorithms may have become a prerequisite for clinical adoption [86].

A large amount of the studies reported in this paper employed CV methods (44,84.6%), especially k-fold CV. CV, which is one input dataset split into parts, some of which are used as training classifiers (training data), and the remainder is used for validation (test data), this method is relatively economical, and could deal with overfitting and generalization problems to a certain extent [87]. However, due to the unbalanced nature of the number of features and the number of subjects in each study, as well as the high heterogeneity of the study sample, the generalization is still limited. Moreover, internal verification cannot guarantee the quality of ML model, it has no extrapolation [87]. Leave-one-out CV (LOOCV) is a special form of k-fold CV, which divides the data set into N subsets (N is the total number of samples). Only 1 sample is retained as the test set each time, and the remaining N-1 samples are repeated for N times. Finally, the average value of all test results is taken as the model evaluation index [28, 88]. This verification method can maximize the data utilization rate and is suitable for capturing the heterogeneity among ADHD individuals (such as the differences in neural markers of different subtypes). However, due to the high computational cost, it is not friendly to multi-modal high-dimensional data (such as fMRI), and can only be used for small data sets [28, 89]. AI requires large datasets to train models in order to avoid over-fitting and improve generalization. Only seven studies used datasets with more than 1,000 data points, and 21 studies used open datasets. In order to reflect the actual performance of the AI model in neuropsychiatric diseases, the model needed to be tested on multiple data sets to ensure its extrapolation [6]. AI models in future should be trained and validated in larger datasets [90]. DL has no advantage over ML in terms of classification and consumes more resources. However, the emergence of DL will further continue in the era of pediatric clinical studies because of its lesser reliance upon the existence of engineered features [91].

### Comparison with previous studies

So far, we had retrieved five reviews on the use of AI in ADHD. A summative review explored the complex interaction of multiple cognitive, genetic and biological factors related to ADHD underling the ML-based algorithm [5]. The authors reported the significance of ML models in ADHD research. Loh, Ooi [92] conducted a systematic review by following PRISMA guidelines and focused on the diagnostic value of AI-based, they identified existing diagnostic tools for ADHD that are commonly used: EEG, MRI, questionnaires, exercise data, performance tests, etc. From the perspective of each diagnostic tool, the most commonly used features were discussed. Pereira-Sanchez and Castellanos [93] provided a brief narrative review of recent AI studies using sMRI and fMRI in ADHD patients, focusing on meta-analyses, large analyses, and proposed novel multimodal approaches. Periyasamy, Vibashan [20] provided a literature review on the application of AI in ADHD. In studies focusing on the use of MRI data, the feature extraction, dimensionality reduction/feature selection, and classification techniques were compared. Taspinar and Ozkurt [19] reported a review focusing on the inclusion of studies using sMRI data. Our scoping review focused on the role of AI techniques in the diagnosis, classification, and prediction of ADHD, following PRISMA guidelines. Provide the purpose and characteristics of all AI technologies listed in the study by reviewing the data sources and platforms used by the AI model. Hopefully, our findings will contribute to further ADHD research.

### Limitations

This study had the following limitations. This review did not include articles related to the prognosis, treatment, and drug discovery of ADHD. The review was limited to journal articles written in English, while papers, review articles, conference abstracts, and review reports were excluded to reduce the complexity of the results. In fact, many research articles in the field of computers are published in full through conferences. In addition to popular public databases, half of the studies used private datasets, there was heterogeneity between studies in the methods and datasets used to generate assessment measures, such as the number of participants, data collection methods, and validation methods used. Finally, we only searched four commonly used databases, and there may have been omissions in some unsearched databases.

## Conclusion

This scoping review is undertaken to support the existing evidence on the role of AI techniques in ADHD. We summarized AI models and algorithms for prediction, early diagnosis, and classification. Research into the application of AI to ADHD is still in its infancy, but early attempts to study ADHD using AI have shown promising results. Translating research into clinical practice still has a long way to go, and more explainable AI research and public education initiatives are needed. We believe that this review will help the scientific community better understand the application of AI techniques in ADHD.
