# Supplementary material for: Artificial intelligence for children with attention deficit/hyperactivity disorder: a scoping review
Source: Exp Biol Med (Maywood). 2025 Apr 24;250:10238. doi: 10.3389/ebm.2025.10238 (PMC12058481; doi:10.3389/ebm.2025.10238)
Supplement: Supplementary file 1 [file Table1.DOCX]

**Appendix 1:** Search Terms used to find studies.

| **Database** | **Hits** | **Search Terms** |
| --- | --- | --- |
| PubMed | 523 | ("artificial intelligence" OR "machine learning" OR "deep learning" OR "supervised learning" OR "unsupervised learning" OR "reinforcement learning") AND (“attention deficit hyperactivity disorder") AND (diagnosis* OR detect* OR predict* OR screen*) |
| BioRXiv | 408 | ("artificial intelligence" OR "machine learning" OR "deep learning" OR "supervised learning" OR "unsupervised learning" OR "reinforcement learning") AND (“attention deficit hyperactivity disorder") AND (diagnosis* OR detect* OR predict* OR screen*) |
| MedRxiv | 99 | ("artificial intelligence" OR "machine learning" OR "deep learning" OR "supervised learning" OR "unsupervised learning" OR "reinforcement learning") AND (“attention deficit hyperactivity disorder") AND (diagnosis* OR detect* OR predict* OR screen*) |
| ScienceDirect | 450 | ("artificial intelligence" OR "machine learning" OR "deep learning" OR "supervised learning" OR "unsupervised learning" OR "reinforcement learning") AND (“attention deficit hyperactivity disorder") AND (diagnosis* OR detect* OR predict* OR screen*) |
