# Supplementary material for: Artificial intelligence for children with attention deficit/hyperactivity disorder: a scoping review
Source: Exp Biol Med (Maywood). 2025 Apr 24;250:10238. doi: 10.3389/ebm.2025.10238 (PMC12058481; doi:10.3389/ebm.2025.10238)
Supplement: Supplementary file 3 [file Table2.DOCX]

**Appendix 2:** Interrater agreement matrices for study selection.

| **Interrater Agreement Matrices** | | | | |
| --- | --- | --- | --- | --- |
| **Title and Abstract Screening** | | | | |
|  | | **Reviewer 1** | | |
|  |  | **Include** | **Exclude** | **Total** |
| **Reviewer 2** | **Include** | 242 | 14 | 256 |
|  | **Exclude** | 8 | 1174 | 1182 |
|  | **Total** | 250 | 1188 | **1438** |
| **Full text Screening** | | | | |
|  | | **Reviewer 1** | | |
|  |  | **Include** | **Exclude** | **Total** |
| **Reviewer 2** | **Include** | 52 | 9 | 61 |
|  | **Exclude** | 15 | 153 | 168 |
|  | **Total** | 67 | 162 | **229** |

Cohen's Kappa (κ) measures inter-rater agreement, calculated as κ = (Po - Pe) / (1 - Pe), based on confusion matrix diagonals for Po and marginal frequency products for Pe.
